# *Talaromyces pinophilus* Strain HD25G2 as a Novel Biocontrol Agent of *Fusarium culmorum*, the Causal Agent of Root and Crown Rot of Soft Wheat

**DOI:** 10.3390/jof11080588

**Published:** 2025-08-11

**Authors:** Amel Bennacer, Fatma Sahir-Halouane, Micaela Alvarez, Zahia Oukali, Nour El Houda Bennacer, Abdelhamid Foughalia, Josué Delgado

**Affiliations:** 1 Laboratory of Valorization and Conservation of Biological Ressources (VALCORE), Department of Biology, Faculty of Sciences, University of M Hamed Bougara, Boumerdes 35000, Algeria; a.bennacer@univ-boumerdes.dz (A.B.); fatmahalouane@yahoo.fr (F.S.-H.); oukaliz408@gmail.com (Z.O.); 2 Grupo de Investigación Higiene y Seguridad Alimentaria, Instituto Universitario de Investigación de la Carne y Productos Cárnicos, Facultad de Veterinaria, Universidad de Extremadura, 10003 Cáceres, Spain; jdperon@unex.es; 3 Sección Departamental de Nutrición y Ciencia de los Alimentos, Facultad de Veterinaria, Universidad Complutense de Madrid, 28040 Madrid, Spain; 4 Laboratory of Water Environment and Sustainable Development, Faculty of Technology, University Blida 1, Blida 09000, Algeria; bennacer.noorelhouda@gmail.com; 5 Centre de Recherche Scientifique et Technique sur les Régions Arides (CRSTRA), BP 1682 RP, Biskra 07000, Algeria; hmadou.fou@gmail.com

**Keywords:** antagonism, enzymatic activity, food security, mycelial growth, zearalenone

## Abstract

*Fusarium culmorum* is the causal agent of root rot and crown rot in soft wheat. The aim of this study was to investigate the control mechanism of *Talaromyces pinophilus* HD25G2 as a biocontrol agent against *F. culmorum*. This involved the isolation and molecular identification of *Fusarium* and *Talaromyces* strains from soft wheat. The assay included the inhibition test of *F. culmorum* mycelial growth on potato dextrose agar and soft wheat media at two water activity values (0.98 and 0.95), its production of mycotoxins, and the fungal cell wall-degrading enzymes implicated in the antagonistic effect of *T. pinophilus*. The results showed that *T. pinophilus* and its extract free of cells reduced the growth of *F. culmorum* by over 55%. Interestingly, the *T. pinophilus* HD25G2 showed high chitinase, protease, and cellulose production on solid media. In addition, chitinolytic and proteolytic activities were estimated at the values of 1.72 ± 0.02UI and 0.49 ± 0.01UI, respectively. However, the mycotoxin evaluation assay revealed that *F. culmorum* HD15C10 produced zearalenone (ZEA) and the biocontrol agent enhanced its production, but the early inoculation of *T. pinophilus*, before *F. culmorum* growth onset, inhibited 100% its growth and, therefore, prevented the presence of ZEA. Hence, this strain can be proposed as a biocontrol agent against *F. culmorum*, and it can be further investigated for biocontrol of *Fusarium* root and crown rot in vivo.

## 1. Introduction

The world’s crop production experiences significant crop losses each year due to pathogens through reduced yield, productivity, and growth of many crops [1]. In this sense, *Fusarium* infection, a plant pathogenic fungus, is a global concern, causing economically severe crop losses and ranking among the most hazardous pathogens affecting cereals [2]. *Fusarium* encompasses a diverse group of fungal species, many of which are well-known plant pathogens that have caused significant crop damage over the past two centuries. These fungi commonly colonise aerial parts of plants, either as natural components of the mycobiota or as aggressive pathogens, particularly affecting horticultural crops and cereal grains like maize, often rendering them unsuitable for consumption. *Fusarium* species are responsible for various plant diseases, including seedling blight, root and crown rot, as well as stalk and ear rot, and can infect plants at any developmental stage. The success of *Fusarium* infection depends on a series of complex and tightly regulated processes, beginning with host colonisation and culminating in disease manifestation [2,3]. Additionally, this genus poses a risk due to its ability to synthesise mycotoxins such as deoxynivalenol (DON), zearalenone (ZEA), and fumonisin B1. These mycotoxins are identified as some of the top five most significant mycotoxins [3]. Fusariosis has a significant economic impact across various countries and regions worldwide, including North and South America, Australia, Europe, North Africa (particularly Algeria), South Africa, and West Asia. Losses can reach up to 70% when infection conditions are favourable. They reduce yield by decreasing the germinative capacity of seeds, the grain count per spike, the weight of a thousand grains, and their overall quality [4]. Investigations have shown that strains of *Fusarium culmorum* are prevalent and highly aggressive on wheat seedlings along with *Fusarium graminearum* and *Fusarium pseudograminearum*, contributing to head blight (FHB), crown rot (FCR) and root rot (FRR) diseases in Algeria [5,6]. These diseases affect soft wheat (*Triticum aestivum*) worldwide, causing substantial yield losses, especially in dry climates and regions where conservation agriculture practices, such as minimal tillage and stubble retention, are applied. FCR is mainly spread through infected stubble [7], FRR is worsened by the use of infected seeds, and FHB is identified by the bleaching of spikelets [8,9]. Aside from causing yield losses, *F. culmorum* also leads to the build-up of diverse mycotoxins, mainly ZEA [10]. To control these hard-to-reach fungi, a range of strategies has been proposed, including crop rotation, the use of resistant varieties, or antifungal treatment. These are the main practical approaches used with limited effectiveness [11]. Plant protection treatments relying on synthetic chemicals are increasingly scrutinised owing to worries over human health, environmental impact, the development of resistant strains, and residue presence in harvested fruit [12]. Therefore, due to *Fusarium* dissemination and its persistence in the soil, its control seems limited to prophylactic measures [13]. However, in recent years, special attention has been given to methods of biological control. The use of microorganisms with antifungal activity is a promising alternative to the use of pesticides, due to the ubiquity of these microorganisms, their great diversity, and their dissemination in rhizospheric soils, and it is potentially beneficial to the health of consumers [14]. Previous research has shown that *Talaromyces pinophilus* (Hedgc.) Samson is prevalent in soil, compost, seeds, grains, the phyllosphere, and the phylloplane. Furthermore, it has been recognised as an endophytic fungus in several therapeutic plants [1,15,16]. It has been widely utilised for its ability to degrade cellulose and as a sustainable source of pigments and bioactive compounds [17,18]. Few studies have looked at *T. pinophilus*’ antagonistic activity and mycoparasitic behaviour against *Rhizoctonia solani* [19,20]. The detailed mechanism of *T. pinophilus* mycoparasitism against *B. cinerea* was outlined by Abdel-Rahim and Abo Elyossr [21]. They concluded that the fungus’s toxic metabolites, including extracellular wall-degrading enzymes, antibiotics, and volatile organic compounds (VOCs), contribute to the expression of its antagonistic activity [22,23].

This research aimed to evaluate the antagonistic effects of *T. pinophilus* on *F. culmorum*, the pathogen is responsible for *Fusarium* Root and *Fusarium* Crown Rot in soft wheat. This study focused on examining the inhibition of *F. culmorum* growth, mycotoxin production, as well as the enzymes that degrade the fungal cell wall, which play a role in the antagonistic interaction. To the best of our knowledge, this is the first study worldwide to investigate these specific antagonistic mechanisms between *T. pinophilus* and *F. culmorum*.

## 2. Materials and Methods

### 2.1. Fungal Isolation and Identification

*Fusarium* sp. HD15C10 was isolated from samples of soft wheat seeds, harvested in the year 2020, from the Tizi-ouzou region (36.73601203227208, 3.9740759227314664) (Algeria), while the *Talaromyces* spp. HD25G2 was isolated from healthy, asymptomatic soft wheat seeds stored in the Constantine region (36.334561393967014, 6.714242306223273), Algeria.

The seed samples (1 Kg) were transferred into sterilised polyethene bags and promptly taken to the laboratory and refrigerated until isolation. Isolation was performed using PDA medium (TM Media, Delhi, India) enriched with chloramphenicol (250 mg/L) and rose Bengal (66.7 mg/L) (both from Sigma-Aldrich, St. Louis, MO, USA).

In order to isolate the fungi, soft wheat seeds from both samples were individually selected, surface-sterilised using 1% sodium hypochlorite, thoroughly rinsed with sterilised distilled water, and subsequently dried on a sterile filter paper. The sterilised seeds (5 grains per plate) were then placed on PDA plates and incubated at 25 °C for 7 days [24].

After incubation, the colonies were subsequently purified through a single-spore isolation technique. The monosporic colonies were further examined both macroscopically and microscopically [25,26].

Macroscopic characterisation involved evaluating morphological features such as colony growth rate, texture, and colour. Microscopic observations of *Talaromyces* spp. and *Fusarium* spp. isolates were carried out by examining the shape of phialides and conidia, as well as the positioning of chlamydospores, using a Motic BA210 model microscope (Fisher Scientific, Schwerte, Germany). The endophytic fungus *Talaromyces* spp. was initially identified through its colony morphology, growth texture, and micromorphological features on potato dextrose agar (PDA), glycerol nitrate 25% agar (GN25) at 25 °C, and Czapek yeast agar (CYA; Sigma-Aldrich) at 37 °C after 7 days of incubation. The fungi were subsequently stored in the laboratory’s mycological collection for future use. The isolation was performed in triplicate.

### 2.2. Molecular Identification of Talaromyces spp. and Fusarium spp. Strains by PCR Assay

Molecular identification of the fungal isolate was achieved through PCR amplification targeting the internal transcribed spacer (ITS) region of ribosomal DNA. Fungal mycelium was harvested from potato dextrose agar (PDA) after 5 days of incubation at 28 °C, suspended in 100 μL of sterile distilled water, and stored at −80 °C. Genomic DNA was extracted utilising the NucleoSpin Plant II DNA Prep Kit (Macherey-Nagel, Düren, Germany), following the SDS/CTAB lysis method, with subsequent purification via phenol/chloroform extraction. PCR amplification and sequencing were performed at GLS Sidi Bel Abbes (Sidi Bel Abbès, Algeria) and Eurofins Scientific (Nantes, France). The ITS region was amplified using the primer pair ITS1 (5′-CTTGGTCATTTAGAGGAAGTAA-3′) and ITS4 (5′-TCCTCCGCTTATTGATATGC-3′), as described by Gardes and Bruns [27].

The PCR reaction mixture, with a total volume of 25 μL, contained 1 μL (20 ng) of fungal DNA, 2.5 μL of 10× EF-Taq buffer, 0.5 μL of 10 mM dNTPs, 1.0 μL of each forward and reverse primer (10 pmol), 0.25 μL of EF-Taq polymerase (2.5 U), and nuclease-free water to complete the volume. The PCR conditions included an initial denaturation at 95 °C for 5 min, followed by 35 cycles consisting of denaturation at 95 °C for 30 s, annealing at 55 °C for 30 s, and extension at 72 °C for 45 s, concluding with a final extension at 72 °C for 7 min. PCR products were visualised on an agarose gel under UV light (VWR-730 Avantor, Radnor, PA, USA). Specific bands were excised and purified using the NucleoSpin Plant II kit (Fisher Scientific, Schwerte, Germany) following the manufacturer’s protocol. The amplified ITS region from both strands was sequenced using the primers ITS1 and ITS4. The resulting sequences were analysed through the BLAST tool on the NCBI website (http://blast.ncbi.nlm.nih.gov/Blast.cgi).

Multiple sequence alignments were performed using CLUSTALW (https://www.genome.jp/tools-bin/clustalw), and phylogenetic relationships were determined using MegAlign software (version 5.01) with reference sequences from GenBank.

The sequences for *T. pinophilus* strain HD25G2 and *F. culmorum* HD15C10 were deposited in the GenBank nucleotide sequence database under accession numbers OM978188 and ON743052, respectively.

### 2.3. Antagonistic Activity

All assays were conducted on PDA and soft wheat simulating media. The 2% milled soft wheat agar media (SWA) was prepared and adjusted at two distinct water activities (a_w_) values by adding glycerol until it achieved 0.98 (high water availability; SWA98) and 0.95 a_w_ (intermediate water stress; SWA95) according to Da Cruz Cabral et al. [28].

#### 2.3.1. Dual Culture Test

A 6 mm diameter mycelial plug of actively growing *F. culmorum* was placed on one edge of a Petri dish containing PDA, SWA98 and SWA95, while a mycelial plug of *T. pinophilus* was placed on the opposite edge, 35 mm away. The plates were incubated at 25 °C until the morphological overgrowth interaction was observed. The pathogen *F. culmorum* was inoculated alone in the middle of the testing plates as a positive control [29]. To calculate the growth inhibition percentage (I%), the following equation was used:

I% = [(RC − RT)/RC] × 100(1)

In this formula, RC denotes the diameter of the pathogen’s radial growth in the control treatment, while RT represents the diameter of radial growth when the pathogen is exposed to the biocontrol agent (BCA).

Additionally, after the results were obtained, an assay of direct confrontation was required. The mycelium close to the contact zone in dual cultures was observed microscopically. All the assays (antagonistic activity and dual culture as well as negative controls) in different media were performed in triplicate.

#### 2.3.2. *Talaromyces pinophilus* Culture Filtrate Inhibition Test

The method involves adding culture filtrates from the antagonistic strain *T. pinophilus* into a culture medium, followed by inoculation with discs of the pathogen to evaluate its effectiveness against *F. culmorum* [28,29]. Three plugs from the *T. pinophilus* culture were transferred to Erlenmeyer flasks containing 150 mL of PDB or SWB (at two different a_w_ levels) and incubated on a rotary shaker at 150× *g* and 26 °C for 7 days. After incubation, the liquid culture was harvested by centrifugation at 9500× *g* for 10 min and subsequently filtered using a 0.45 μm syringe filter (RephiLe Bioscience, Philadelphia, PA, USA). A negative control was prepared, maintaining the same conditions. The resulting filtrates from PDB and SWB were incorporated into PDA and SWA98 or SWA95 media, respectively, at a 10% (*v*/*v*) concentration. Three 6 mm diameter agar plugs from the *F. culmorum* culture were placed at equal distances (35 mm apart) on the Petri dish, which was then incubated at 26 °C until the fungal colonies overlapped. The inhibition percentage (I%) was determined using the method described earlier.

### 2.4. Direct Confrontation Test

This test was used to evaluate the antagonistic interaction between BCA mycelium and *F. culmorum* mycelium in SWA98 and SWA95 media in direct contact, applying the BCA before the growth of *F. culmorum* [30,31]. In SWA plates at the two a_w_, 100 µL of *T. pinophilus* strain (10^Q^ spore/mL) was inoculated on all the surfaces and incubated at 25 °C. After three days, a mycelium plug (6 mm diameter) from 4-day-old actively developing colonies of *F. culmorum* was seeded in the centre of the plates. The plates were observed daily (9th day after incubation). Negative controls of both strains were grown separately. The assays in different culture media, as well as the negative control, were conducted in triplicate.

### 2.5. Mycotoxin Production

Three plugs from *F. culmorum* mycelia close to the contact zones between moulds from the dual culture test in SWA media (Section 2.3.1) were collected. The mycotoxins were extracted and analysed following the methodology described by Galán et al. [31]. Briefly, acetonitrile, water, and acetic acid (79:20:1; *v*/*v*/*v*) were added to the sample at a ratio of 4 mL/g, and the solution was shaken for 90 min. Following extraction, a 0.5 mL aliquot of the extract was mixed with 0.5 mL of a different acetonitrile/water/acetic acid solution in a 20:79:1 (*v*/*v*/*v*) ratio and transferred into 1.5 mL vials. A 10 μL portion of the diluted extract was subsequently analysed using target metabolomics in a Q-Exactive Plus Orbitrap mass spectrometer (Thermo Fisher Scientific, Waltham, MA, USA).

### 2.6. Enzymes Qualitative Screening of T. pinophilus HD25G2

Protease, chitinase, and cellulase activities were evaluated on skim milk agar, Chitinase detection Medium (CDM), and Czapek-Mineral Salt agar medium supplemented with carboxy methyl cellulose (CMC). All the media cultures were maintained at pH 7 and incubated in the dark as described by Cherukupally et al. [32] and Reghmit et al. [33]. The results are observed according to the change in the media’s colour:

- Clear zones around the disc indicate proteolytic activity.
- The chitin hydrolysis area changes colour from yellow to purple.
- The presence of a yellow opaque zone surrounding the colonies shows the presence of cellulases.

### 2.7. Quantitative Enzyme Assays

#### 2.7.1. Determination of Protease Activity

To evaluate proteolytic activity, a liquid medium supplemented with 1% casein as the substrate was used, following the method described by Reghmit et al. [33]. The cultures were grown for 5 days at 28 °C, after which they were centrifuged at 9500× *g* for 15 min.

A 500 µL aliquot of the supernatant was then mixed with 1.25 mL of 1% casein prepared in phosphate buffer (pH 10) and incubated at 40 °C for 30 min. The reaction was terminated by adding 3 mL of 0.19 M trichloroacetic acid (Sigma-Aldrich), followed by a further incubation at 40 °C for 15 min. The mixture was then subjected to centrifugation at 9500× *g* for 10 min.

After the centrifugation process, 500 µL of the supernatant was combined with 2.5 mL of 0.4 M NaCO_3_ and 250 µL of Folin–Ciocalteu’s reagent (Sigma-Aldrich). The resulting solution’s absorbance was measured at a wavelength of 660 nm to determine the concentration of released short peptides and free amino acids. Subsequently, enzyme activity was calculated based on the amount of enzyme required to release 1 µmol of tyrosine per minute, per mL of enzyme solution. A negative control (without inoculating the fungi) was also prepared, maintaining the same conditions.

#### 2.7.2. Chitinase Hydrolytic Activity Assay

Following the method described by Reghmit et al. [33]. Two plugs from a 5-day-old *Talaromyces* culture were transferred into 50 mL of synthetic medium (SM). The culture was incubated and then centrifuged at 8000× *g* for 5 min. The supernatant, which served as the enzyme solution, was mixed in equal volume (1 mL) with 1% colloidal chitin prepared in 0.05 M sodium acetate buffer (pH 5). This mixture was incubated for 1 h at 37 °C under constant agitation. After the incubation, the reaction mixture underwent a second centrifugation at 5000× *g* for 15 min. To assess chitinase activity, 1 mL of the supernatant was mixed with 3 mL of dinitrosalicylic acid (DNS) reagent and boiled for 5 min. The absorbance of the solution was recorded at 585 nm. Chitinase activity was expressed as the amount of enzyme needed to release 1 µmol of N-acetylglucosamine (GlcNAc) per minute under the assay conditions. All experiments were performed in triplicate. A negative control (without inoculating the fungi) was also prepared, maintaining the same conditions.

### 2.8. Statistical Analysis

Values were analysed using two-way ANOVA with Tukey’s HSD to study the interactions between the different media and treatments on growth inhibition and mycotoxins due to within the natural population, biological data generally follow a normal distribution. All the tests were carried out using IBM SPSS software version 25.0 (IBM Corp., Armonk, NY, USA). Cohen’s term *d* was used to calculate the effect size index by using R software version 4.5.1. (https://www.r-project.org). Statistical significance was set at *p* ≤ 0.01.

## 3. Results

### 3.1. Fungal Isolation and Identification

**Figure 1:**
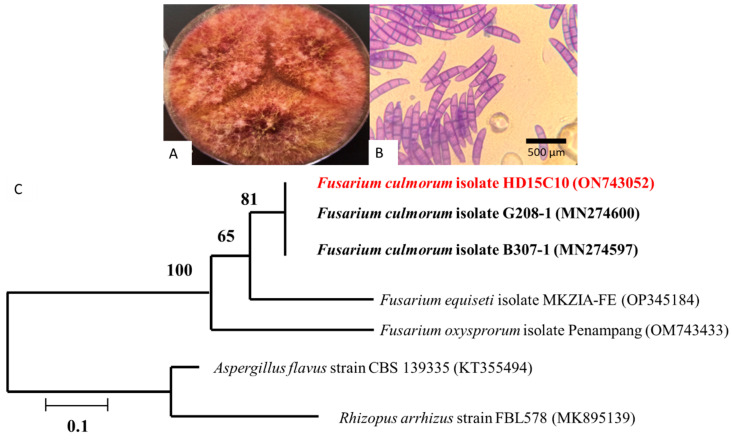
*Fusarium culmorum* strain HD15C10: (**A**) 7-day-old culture at 25 °C on potato dextrose agar (PDA) medium; (**B**) light microscopic analysis showing the fungal elements as conidiophores and conidia; (**C**) molecular phylogeny of *F. culmorum* strain HD15C10 with DNA sequences for reference strains of *F. culmorum* (bar indicates genetic distance due to sequence divergence).

In this study, *F. culmorum* HD15C10 was isolated from soft wheat seed crops grown in Tizi-Ouzou, Algeria. Preliminary *F. culmorum* strain HD15C10 characterisation was carried out based on macroscopic colony traits and microscopic features. The following figure demonstrates the macroscopic and microscopic appearances (Figure 1A,B) and, in addition, the molecular phylogeny of *F. culmorum* strain HD15C10 with DNA sequences for reference strains (Figure 1C).

Sequencing of the ITS region showed 99.4% similarity with *F. culmorum* isolate G208-1 (MN274600), and a phylogenetic tree was constructed using other closely related sequences of *F. culmorum* (Figure 1C).

**Figure 2:**
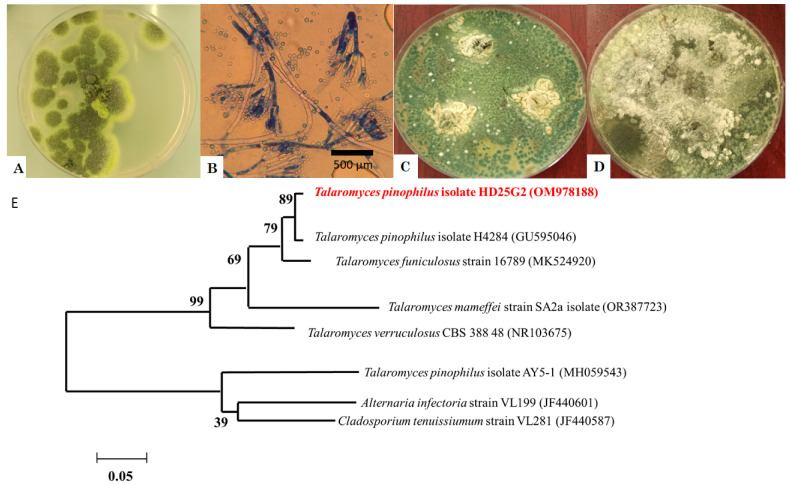
*Talaromyces pinophilus* strain HD25G2: (**A**) 7-day-old culture on PDA medium at 25 °C; (**B**) conidiophores with smooth walled elements, characteristically symmetrically biverticillate; (**C**) 7-day-old culture on GN25 medium at 25 °C; (**D**) 7-day-old culture on CYA medium at 37 °C; (**E**) molecular phylogeny of *T. pinophilus* strain HD25G2. with DNA sequences for reference strains of *Talaromyces* spp. (bar indicates genetic distance due to sequence divergence).

Figure 2 represents the macroscopic and microscopic appearances (Figure 2A–D) and the molecular phylogeny of *T. pinophilus* HD25G2 with DNA sequences for reference strains (Figure 2E).

On PDA, colonies of *T. pinophilus* grew to 27–32 mm after 7 days of incubation at 25 °C, displaying a greyish-green to dull green colour with white and yellow mycelia (Figure 2A). When cultured on CYA and GN25 media, after 7 days of incubation at 37 °C (Figure 2C) and 25 °C (Figure 2D), the colonies appeared greyish-green, with a raised centre and flat, well-defined edges. The texture was floccose and funiculose, particularly in the central region (Figure 2A). The reverse side of the colonies exhibited a greyish-orange colouration. Conidiophores were smooth, symmetrically biverticillate, with stipes (150–200 × 2–3 μm) bearing three to six metulae (10–11 × 2.5–3 μm), which supported acerose phialides (8.5–12 × 2–3 μm) (Figure 2B). The conidia were smooth, globose to subglobose, with a diameter ranging from 2 to 3 μm. No ascomata were observed. Additionally, the ITS sequence of the *T. pinophilus* strain HD25G2 showed 99.5% similarity with *T. pinophilus* isolate H4284 (GU595046) (Figure 2E).

### 3.2. Mycelial Growth Inhibition

**Figure 3:**
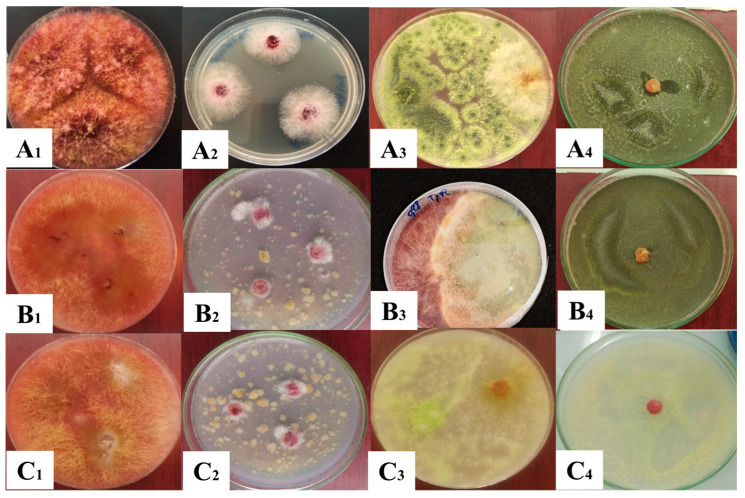
(**A_1_**–**A_4_**) Assays on potato dextrose agar media (image A_1_ also shown in Figure 1A), (**B_1_**–**B_4_**) on soft wheat agar media a_w_ 0.98, and (**C_1_**–**C_4_**) on soft wheat agar media a_w_ 0.95 (1: *Fusarium culmorum* control, 2: filtrate assay, 3: direct confrontation assay, 4: direct confrontation (*Talaromyces pinophilus* was cultivated 2 days before *F. culmorum*)).

The biocontrol potential of the *T. pinophilus* isolate was in vitro assessed through different mechanisms against *F. culmorum*. The inhibition effect of *T. pinophilus* on the mycelial growth of *F. culmorum* in dual culture assay displayed a percentage of inhibition around 72.59% (PDA), 73.7% (SWA98), and 72.01% (SWA95) after 7 days of incubation (Figure 3) compared to the untreated control (showing no activity) (*p* < 0.01; Table 1). The results demonstrated a large effect size when *T. pinophilus* was added concerning the untreated control, with Cohen’s *d* values ranging from 7 to 21. No statistical differences were found between the same treatments in different media after the analyses using two-way ANOVA with Tukey’s HSD (*p* > 0.01). Cohen’s *d* values between media were in the range of −0.4 and 0.2 in the dual culture assay and between 0 and 0.1 in the filtrate culture. Importantly, BCA was most efficient in blocking 100% mycelial growth of *F. culmorum* HD15C10 when administered 3 days after the BCA in the direct confrontation test (Figure 3(A_4_,B_4_,C_4_)).

The filtrate of *Talaromyces* demonstrated the ability to inhibit the growth of the pathogen (Table 1). This strain effectively restricted both radial and aerial growth. The results suggest that the inhibition percentage is likely influenced by the concentration of *Talaromyces* filtrate and the quality of the bioactive molecules present (Figure 3(A_2_,B_2_,C_2_)).

**Figure 4:**
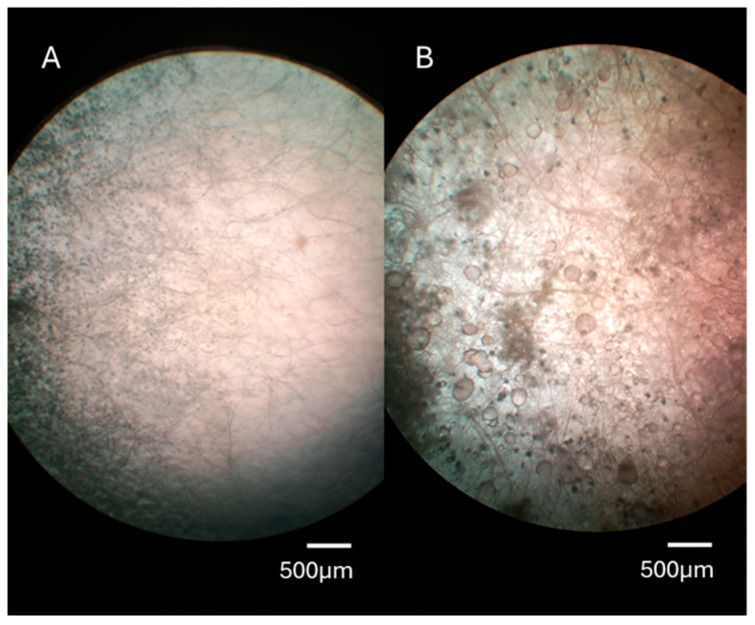
Microscopic evaluation of *Fusarium culmorum* HD15C10. (**A**): Normal growth of *F. culmorum* in soft wheat agar with 0.98 water activity (a_w_). (**B**): *F. culmorum* growth close to the contact zone with *Talaromyces pinophilus* used as a biocontrol agent.

The microscopic evaluation of *F. culmorum* in contact with *T. pinophilus* is represented in Figure 4. The microscopical visualisation showed a denser mycelium from *F. culmorum* in the confrontation zone with the BCA (Figure 4B) with respect to the normal growth of the pathogen (Figure 4A). Furthermore, there was an increase in the exudate droplet production by the toxigenic mould when confronted with the BCA (Figure 4).

### 3.3. Mycotoxin Analysis

The analysis revealed that *F. culmorum* HD15C10 is a producer of ZEA. The results (Table 2) showed that the presence of the BCA triggered the ZEA production. Cohen’s *d* showed a large effect size when adding the biocontrol agent (−3.40 at 0.95 a_w_ and −32.55 at 0.95 a_w_).

### 3.4. T. pinophilus Extracellular Enzymes

**Figure 5:**
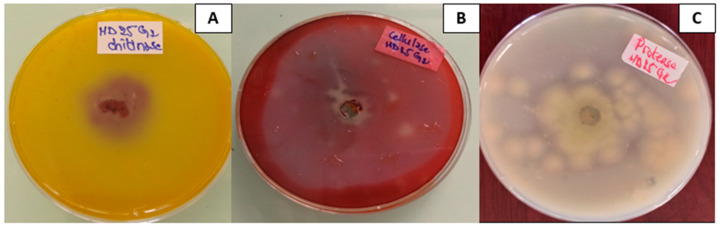
*Talaromyces pinophilus* HD25G2 production of extracellular enzymes on selective solid medium. Positive enzymatic activity indicated by a change in colour of the medium around the colony. (**A**): chitinase activity; (**B**): cellulase activity; (**C**): protease activity.

Figure 5 represents the appearance of *T. pinophilus* on solid media for the enzymatic activity assays indicated by clear zones around the colony. The tested *T. pinophilus* strain showed the capacity to produce chitinases, proteases, and cellulases. The strain showed cellulase activity, as indicated by an 80 mm clear zone around the colonies after 3 days of incubation. It also exhibited notable protease activity, demonstrated by visible protein hydrolysis on skim milk agar (clear zone). Additionally, *T. pinophilus* displayed strong chitinase activity, evidenced by a purple zone with a diameter of 65 mm (Figure 5). Enzyme assays revealed that the proteolytic activity of *T. pinophilus* was 0.49 ± 0.01 IU at pH 10, while its chitinase activity was 1.72 ± 0.02 IU compared to negative controls that showed no enzymatic activity.

## 4. Discussion

Significant efforts have been made to replace chemical fungicides with environmentally friendly alternatives, particularly biological control agents (BCAs) [34,35]. A critical first step in biological control is the identification of effective antagonistic agents. However, research on the antagonistic potential of *T. pinophilus* as a BCA against phytopathogenic fungi remains limited [17,20,21]. Endophytes are microorganisms that reside within the tissues of plants that appear healthy and show no symptoms of infection [36].

To the best of our knowledge, this is the first study to investigate the endophytic fungus *T. pinophilus* as a biological control agent (BCA) against *F. culmorum*. Notably, the *T. pinophilus* strain HD25G2 was predominantly isolated from healthy, asymptomatic stored soft wheat seeds from the Constantine region in Algeria.

This study demonstrated for the first time the inhibition of *F. culmorum* mycelial growth by *T. pinophilus*. Dual culture techniques have been widely used in tests of antagonistic activities, showing useful in vitro results [33,37], such as those in Figure 3. Previous studies have indicated that *Talaromyces* species are commonly found in soil, air, and the seeds of various plants, with some strains showing potential for controlling plant diseases [38]. For instance, *Talaromyces flavus*, isolated from tomatoes, significantly reduced tomato blight caused by *Verticillium dahlia* [39]. Similarly, the Q2 strain of *T. purpureogenus* has demonstrated the ability to suppress the growth of 12 plant pathogens and mitigate soil-borne diseases, including bitter melon wilt, tobacco black shin disease, and potato stem rot, under controlled greenhouse conditions [40]. *T. muroii* TM28 demonstrated effective inhibition of *Fusarium pseudograminearum*, with an inhibition rate exceeding 50% [41]. Moreover, *T. pinophilus* has also been reported to exhibit inhibitory effects against *B. cinerea*, demonstrating mycoparasitic activity [21]. The sterile fermentation filtrate of *T. pinophilus* demonstrated a high inhibitory capacity on the mycelial growth of *F. culmorum* when cultured on both PDA and SWA media. This observed antagonism is likely attributed to the strain’s production of various cell wall-degrading enzymes and catalytic activities (Figure 4). Previous research has recognised *Talaromyces* species as significant cellulose-degrading fungi, contributing significantly to biomass degradation processes [42,43]. These fungi possess a wide range of genes involved in encoding proteases, enzymes for secondary metabolite biosynthesis, and enzymes that degrade fungal cell walls [44,45,46]. The fungal cell wall is integral to several key processes, including fungal survival, morphogenesis, and pathogenicity [47], and its breakdown is a central mechanism through which antagonistic microorganisms exert their inhibitory effects [48]. Chitin and β-(1,3)-glucan, both critical components of the *Fusarium* cell wall, are synthesised by the enzymes chitin synthase and β-(1,3)-glucan synthase [49]. Chitin plays an essential role in cell division and contributes to the pathogen’s virulence [50]. *T. pinophilus* can produce chitinase, which appears to play a central role in the degradation of the *F. culmorum* cell wall. Previous studies have shown that *T. pinophilus* generates two types of chitinase enzymes, which have been demonstrated to degrade the cell walls of *Verticillium dahliae* and *Rhizoctonia solani*, as well as inhibit spore germination and germ tube elongation of *Alternaria alternata* [51]. However, chitinase alone is not solely responsible for fungal cell wall degradation; it operates synergistically with other hydrolytic enzymes, such as 1,3-β-glucanase, proteases, and lipases, to comprehensively dismantle the host cell wall [52]. In this context, the robust enzymatic activity exhibited by various mycoparasitic organisms has been recognised as a critical factor for the lysis of cell walls in phytopathogenic fungi [53]. In the present study, the *T. pinophilus* strain significantly impacted the mycelial growth of the phytopathogen *F. culmorum*. This finding is consistent with the work of Abdel-Rahim and Abo-Elyousr [21], who documented the mycoparasitic interaction of *T. pinophilus* with *B. cinerea.* A typical mycoparasitic interaction involves several stages, including host detection, attraction, attachment, coiling around the host, and the lysis of the host cell wall through the action of hydrolytic enzymes [54]. Additionally, it has been reported that diffusible compounds, proteins, or antibiotics produced by fungal strains can inhibit the growth of other microorganisms [55,56].

However, despite the successful growth inhibition observed, the BCA stimulated the ZEA production by *F. culmorum* when no total inhibition was achieved (aw 0.98: 114.19 and aw 0.95: 334.16 ng/g compared to the control, *p* ≤ 0.01), demonstrating that the growth measure is not a proper indicator of the effectiveness of BCAs against toxigenic moulds [57,58]. The microscopic evaluation revealed that the mould morphology was also affected and could trigger the production of mycotoxins due to the stress caused by the presence of *Talaromyces*. Several studies have shown that in the presence of stress conditions, such as BCAs, provoking the inhibition of the pathogen’s mycelial growth, the latter could boost the production of mycotoxins, directly affecting the genes responsible for the mycotoxin synthesis [59,60,61].

The ZEA overproduction at a common temperature on field and storage (25 °C) could lead to the discard of *T. pinophilus* as BCA. However, the early inoculation with the BCA prevented the appearance of the toxigenic mould. Therefore, the strategy to successfully counteract *F. culmorum* growth in soft wheat would be the inoculation of this BCA before any possibility of the toxigenic fungal growth onset. This strategy could be exerted both by inoculating crops before harvesting, since this strain is ubiquitous in this environment, or immediately after harvesting, to endow the soft wheat with BCA spores in case it is contaminated with the pathogen and the conditions are propitious for fungal growth. However, this further research step must be carried out in future studies to achieve the best strategy to prevent ZEA accumulation in soft wheat. The stimulation of mycotoxin production has been previously demonstrated in other toxigenic moulds using biocontrol agents and chemical fungicides at subinhibitory concentrations in different matrices. Therefore, the importance of proper application of antifungal agents and the possible need to reinoculate the crops is emphasised by the results of the present study [55,57,62,63].

Efforts should be focused on basic preventative measures such as the correct rate (%) of humidity, prevention of contact with soil, etc. However, the proposed strategy could be a cheap and easy preventative measure for developing countries to avoid the waste of soft wheat in case conditions favour fungal growth, and this wheat could be used for different purposes, such as animal feeding, or destination of the wheat for flour since the fungal growth would not mean mycotoxin production. This alternative would benefit the food safety and security of developing countries, given that it would prevent mycotoxin contamination and food waste. The present study was designed as an exploratory and hypothesis-generating investigation, aiming to provide preliminary insights into the biocontrol potential of *T. pinophilus*, a strain that, to our knowledge, has not been previously evaluated in this specific context. To this end, we employed laboratory-based antagonism assays, enzymatic activity profiling, and co-culture mycotoxin quantification as initial screening tools to assess its potential efficacy. The use of parametric tests in this study is justified by the expectation that, within the natural population, such biological data generally follow a normal distribution, offering increased statistical power and precision. Given the early-stage nature of this work and the absence of data under agronomic or storage conditions, no specific recommendations for field application or commercial formulation can be proposed at this point yet.

## 5. Conclusions

In conclusion, this study provided evidence that the endophytic *T. pinophilus* holds potential as an effective BCA against *F. culmorum*, a producer of zearalenone (ZEA). The production of cell wall-degrading enzymes by *T. pinophilus* enhances its suitability as BCA, offering a promising alternative to chemical fungicides for managing fungal pathogens. The study also confirms that the mycotoxin production of toxigenic moulds must be evaluated when BCA is applied, although it reduces the growth of the pathogens, but does not fully inhibit them. Finally, the use of *T. pinophilus* as a BCA, being a cheap and easy preventive measure, may be proposed for crops with a high probability of suffering conditions that are prone to mould growth, although further research is necessary to measure the best strategy to minimise ZEA contamination. This strategy would positively impact the reduction in mycotoxin contamination and food waste.

## Figures and Tables

**Table 1:** Assessment of antagonistic activity of *Talaromyces pinophilus* HD25G2 against *Fusarium culmorum* HD15C10 on potato dextrose agar and on soft wheat stimulating media at two water activity (a_w_) values.

Media	Assay	Percentage of Growth Inhibition of *F. culmorum* (%)
Potato dextrose agar	Dual culture	72.59 ± 0.37 *^a^
	Filtrate culture	61.48 ± 0.74 *^a^
Soft wheat simulating media a_w_ 0.98	Dual culture	73.7 ± 0.37 *^a^
	Filtrate culture	62.96 ± 3.70 *^a^
Soft wheat simulating media a_w_ 0.95	Dual culture	72.22 ± 2.12 *^a^
	Filtrate culture	59.99 ± 1.03 *^a^

* Statistical differences with the control group (*p* < 0.01). Statistical differences between the same treatment with different media indicated by letters.

**Table 2:** Mycotoxin production of *Fusarium culmorum* HD15C10 in soft wheat agar at 0.98 and 0.95 water activity in the presence of the biocontrol agent *Talaromyces pinophilus* HD25G2.

Water Activity (a_w_)	Treatment	Zearalenone (ng/g)
0.98	Control	0.63 ± 0.84 ^a^
	*F. culmorum* + *T. pinophilus*	104.19 ± 60.98 ^b^
0.95	Control	8.33 ± 6.99 ^c^
	*F. culmorum* + *T. pinophilus*	334.16 ± 17.76 ^d^

Statistical differences are indicated with different letters *p* ≤ 0.01.

## Data Availability

The original contributions presented in the study are included in the article. Further inquiries can be directed to the corresponding author.
